# Neurodynamic regimes of phase relation and behavior in robotic models

**DOI:** 10.1186/1471-2202-12-S1-P155

**Published:** 2011-07-18

**Authors:** Bruno A Santos, Xabier E Barandiaran, Phil Husbands

**Affiliations:** 1 CCNR - University of Sussex, Brighton UK; 2 CREA - Polytechnique/CNRS, Paris, France; 3 LSI, CEFET-MG, Belo Horizonte, Brazil

## 

**Figure 1:**
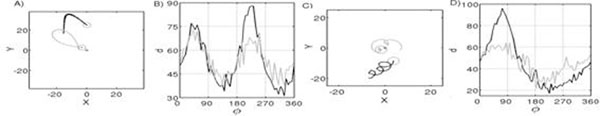
**A** and **C**: two trajectories (black and gray) of the agent's behaviour (circle is the agent's body at the end of each trajectory; light source is positioned at X=0; Y=0). **B** and **D**: show the existence of different dynamical regimes of phase relation for each trajectory (black and gray); ϕ (x-axis) is the phase relation and d (y-axis) is the phase density within the time interval of each trajectory. **B** characterizes 2 different phase regimes which mainly differ by a greater tendency of the black one (which is related to the black trajectory in **A**) to be within [180, 270]. **D** characterizes 2 different phase regimes which mainly differ by a greater tendency of the black one (which is related to the black trajectory in **C**) to be within [70, 90].

Both oscillatory phenomena and the dynamics of integration and segregation in the brain are fields of intense study in current computational neuroscience. It is often suggested that moments of phase-locking (as moments of integration) between functionally distinct neuronal groups represent meaningful functional brain states; and moments of phase-scattering (as moments of segregation) represent transitions between such states. The main evidences for such claim come from investigations on the well-known visual binding problem [1]. Despite the evidences supporting a functionally privileged status for phase locking integration (as a neural signature of cognitive brain activity) an adequate balance between integration and segregation has also been considered essential [2]. We explore how, rather than mere phase-locking, the global *regime of phase relation* might become necessary to generate functional behaviour, i.e. the whole phase dynamic which might include moments of integration (by phase-locking) and segregation (by phase scattering). Our proposal to investigate the whole phase relation dynamics is motivated by the phenomenon of *relative coordination* observed in the movements of fish fins described by von Holst [3] and latter developments of Haken and Kelso's approach to coordination dynamics [4]. In dynamical system terms, by “relative coordination” von Holst means the situation when the phase relation among components is constantly moving in a transient dynamics where some regions of the phase space have low potential energy (moments of phase-locking) and others high potential energy (moments of phase-scattering). Using a genetic algorithm we optimized the parameter of two robotic models performing phototaxis. In one model the agent is controlled by the extended-HKB equation [4] (Fig. 1-A-,1B) and in the other one by a network of five coupled Kuramoto oscillators [5] (Fig. 1-C,1D). Both types of controllers where chosen as paradigmatic of oscillatory modeling in computational neuroscience.

Due to the simplicity of the model we were able to analyze the whole phase dynamic and found different dynamical regimes of phase relation (expressed in terms of phase density) and their behavioral correlates. Despite the strong simplifying assumptions involved, the theoretical results shown here suggest a “different/new” approach to analyze empirical data in neuroscience where regimes of phase relation might be as relevant as phase locking events for how brain-environment dynamics give rise to behaviour. We conclude with some expansions of phase density and phase coherence measurements for bigger oscillatory networks in sensorimotor systems.
